# ZBED6 regulates *Igf2* expression partially through its regulation of *miR483* expression

**DOI:** 10.1038/s41598-021-98777-0

**Published:** 2021-09-30

**Authors:** Rakan Naboulsi, Mårten Larsson, Leif Andersson, Shady Younis

**Affiliations:** 1 Science for Life Laboratory, Department of Medical Biochemistry and Microbiology, Uppsala University, 751 23 Uppsala, Sweden; 2 Department of Animal Breeding and Genetics, Swedish University of Agricultural Sciences, 750 07 Uppsala, Sweden; 3 Department of Veterinary Integrative Biosciences, Texas A&M University, College Station, TX 77843 USA; 4 Department of Animal Breeding and Genetics, Ain Shams University, Shoubra El-Kheima, Cairo, 11241 Egypt; 5 Division of Immunology and Rheumatology, Department of Medicine, Stanford University, Stanford, CA 94305 USA

**Keywords:** Transcriptomics, Non-coding RNAs

## Abstract

The expression of *Igf2* in mammals shows a complex regulation involving multiple promoters and epigenetic mechanisms. We previously identified a novel regulatory mechanism based on the interaction between the transcriptional factor ZBED6 and *Igf2* intron. Disruption of the ZBED6-*Igf2* interaction leads to a dramatic up-regulation of IGF2 expression postnatally. In the current study we characterize an additional layer of regulation involving *miR483* encoded by another *Igf2* intron. We found a highly significant up-regulation of *miR483* expression when the ZBED6-*Igf2* axis is disrupted in transgenic mice. Furthermore, CRISPR/Cas9 mediated knock-out of *miR483* in C2C12 myoblast cells, both wild-type and cells with disrupted ZBED6-*Igf2* axis (*Igf2*^dGGCT^), resulted in down-regulation of *Igf2* expression and a reduced proliferation rate. This was further validated using *miR483* mimics and inhibitors. RNA-seq analysis revealed a significant enrichment of genes involved in the PI3K-Akt signaling pathway among genes down-regulated in *miR483*^−/−^ cells, including *Igf2* down-regulation. The opposite pattern was observed in *Igf2*^dGGCT^ cells, where *Igf2* is up-regulated. Our data suggest a positive feedback between *miR483* and *Igf2* promoter activity, strongly affecting how ZBED6 controls *Igf2* expression in various cell types.

## Introduction

Insulin-like growth factor 2 (*Igf2*) is paternally expressed in mammals and is a crucial fetal growth factor^1^. Transcriptional regulation of *Igf2* is complex and occurs at different levels starting from its genomic locus, which includes the *H19* gene, an imprinted control region (ICR), and a set of enhancers. On the maternal allele, the unmethylated ICR restricts the access of enhancers to the *Igf2* gene, which is therefore silenced, and directs them to promote *H19* expression. In contrast, the heavily methylated ICR on the paternal allele promotes *Igf2* transcription and leads to the corresponding silencing of *H19* expression^1,2^.

Genetic studies in domestic pigs revealed that *Igf2* causes a major Quantitative Trait Locus (QTL) affecting muscle growth and fat deposition^3^, demonstrating that IGF2 also plays an important role for postnatal growth. The identification of a single base change in *Igf2* intron 3 of pigs as the causal mutation of this QTL led to the discovery that this mutation disrupts the binding site for a previously unknown transcription factor, ZBED6^4^. ZBED6 is a transcriptional repressor with the specific binding motif 5′-GCTCGC-3′, present in an intron of *Igf2* and at thousands of other putative binding sites in the mammalian genome^4–8^. Recently, we generated *Zbed6* knock-out mice (*Zbed6*-KO) as well as *Igf2* knock-in mice (*Igf2*-KI) carrying exactly the same single base change found in mutant pigs^9^. Both lines showed a > tenfold upregulation of *Igf2* expression at the mRNA level in skeletal muscle, heart and kidney, an eightfold upregulation of circulating IGF2 and increased muscle growth and enlarged heart, kidney and liver^9^.

*Igf2* shows upregulated expression in both pediatric tumors such as Wilm’s tumor, hepatoblastoma and rhabdomyosarcoma, and adult tumors including colorectal cancer, hepatocellular carcinoma and breast cancer^10^. Loss of imprinting and biallelic expression of *Igf2* is a common finding in tumors. The overexpression of *Igf2* in these tumors are usually much higher than can be explained by loss-of imprinting only. However, the association to *miR483*, a gene hosted in an intron of *Igf2* and co-expressed with *Igf2,* plays a prominent role for enhanced *Igf2* expression in tumors^11^. miR483-3p and miR483-5p, the two miRNA products of *miR483*, are overexpressed in many cancers^11,12^. The transcription of *miR483* is activated by the transcriptional activator USF1^10,13^ that acts as a mediator between the oncoprotein β-catenin (CTNNB1) and the *miR483* locus^14^ in a glucose-dependent manner ^10^. Moreover, miR483 can self-regulate its own expression by indirectlyenhancing the expression of USF1, which in turn stabilizes the transcriptional complex on the *miR483* locus^14^. miR483-3p was found to target DLC-1, BRCA-1 and PUMA which are a candidate tumor repressor, a tumor repressor and an apoptosis modulator, respectively^11,15,16^. On the other hand, miR483-5p was found to increase tumorigenesis^17^ and to be an accurate marker to categorize certain tumors as benign or malignant^18^. Interestingly, it has been reported that miR483-5p binds directly to the 5’UTR region of *Igf2* and thereby enhances *Igf2* transcription^17^.

Here, we explore the functional significance of ZBED6 by a miRNA-seq screen using our *Zbed6*-KO and *Igf2*-KI mice to assess whether ZBED6 regulates miRNA in general and *miR483* in particular. Furthermore, we investigate the role of *miR483* in regulating *Igf2* expression by knocking it out from *Igf2*^+/+^ and *Igf2*^dGGCT^ C2C12 mouse myoblasts.

## Materials and methods

### Animals and tissues

In the current study we used a mouse model that lacks ZBED6 expression (KO) and another mouse model carrying a single base change (G to A) disrupting the ZBED6 binding site at the paternal allele of *Igf2*^*pA/mG*^ (p = paternal, m = maternal)^9^. The pedigree based on mating male *Zbed6*-KO/WT, *Igf2*^*pA/mG*^ and female *Zbed6*-KO/WT, *Igf2*^*G/G*^ mice was established previously^9^. From this cross, *Zbed6-*WT/WT, *Igf2*^*G/G*^ (referred to as WT), *Zbed6*-KO/KO, *Igf2*^*G/G*^ (referred to as *Zbed6*-KO) and *Zbed6-*WT/WT, *Igf2*^*pA/mG*^ (referred to as *Igf2*-KI) mice were generated. WT, *Zbed6*-KO and *Igf2*-KI litter mates were anesthetized at the age of 20 weeks by injecting 16 µL/g body weight with 2.5% Avertin (2,2,2-tribromo ethanol and tertiary amyl alcohol). Skeletal muscle, kidney and liver tissues were collected and saved in RNA-later (Sigma, St. Louis, Missouri, United States).

Mice were kept at Uppsala University and Karolinska Institute. The experimental work was approved by the Uppsala Ethical Committee on Animal Research (#C63/15 and #C143/15) and the Stockholm Ethical Committee (#N38/15). The rules of the Swedish Animal Welfare Agency were followed, and were in compliance with the European Communities Council Directive of 22 September 2010 (2010/63/EU). The study was carried out in compliance with ARRIVE guidelines.

### Total RNA extraction from mouse tissues and miRNA-seq

Total RNA including miRNA were extracted from skeletal muscle, kidney and liver from WT, *Igf2*-KI and *Zbed6*-KO mice. Extractions were done using the miRNeasy mini kit (Qiagen, Hilden, Germany). Quality control and estimates of concentration of the extracted RNA samples were done using TapeStation 2200 (Agilent Technologies, Santa Clara, California, United States) and a NanoDrop ND-1000 spectrophotometer (Thermo Fisher Scientific, Waltham, Massachusetts, United States). miRNA sequencing was done (Center for Genomic Regulation, Barcelona, Spain) for three samples per tissue for each of the three genotypes (total of 27 samples) on a HiSeq system (Illumina, San Diego, California, United States) generating 8 to 16 million 50-bp single-end reads per sample.

### Cell culture, *Igf2*^dGGCT^ and *Igf2*^+/+^ C2C12 myoblasts

*Igf2*^dGGCT^ mouse C2C12 myoblast cells were previously generated using CRISPR/Cas9 and involved a deletion of the nucleotides “GGCT” from the intronic ZBED6 binding site in *Igf2*^19^. The *Igf2*^dGGCT^ and *Igf2*^+/+^ cells were cultured in Dulbecco's Modified Eagle Medium (DMEM) complemented with 10% heat-inactivated fetal bovine serum, penicillin (0.2 U/mL), streptomycin (0.2 µg/mL) and l-glutamine (0.2 µg/mL) (Gibco, Waltham, Massachusetts, United States). Cells were incubated at 37 °C and 5% CO_2_ and were split upon reaching 80–90% confluency. Cells were tested for mycoplasma infection using MycoAlert mycoplasma detection kit (Lonza, Basel, Switzerland) and were shown to be free of infection.

### Generation of *miR483*^−/−^ cells using CRISPR

Guide RNA sequences targeting the mouse *miR483* gene were designed using CRISPRdirect^20^. Two guide-RNA sequences with scores above 90 were chosen. Four single-stranded DNA oligos (Supplementary Table 1) were synthesized (IDT-DNA, Coralville, Iowa, United States), annealed by mixing the forward and reverse oligonuclotides in a 1:1 ratio, heated to 95 °C and allowed to cool down to room temperature at a speed of 0.5 °C/min. Annealed oligonuclotides were cloned into the pSpCas9(BB)-2A-GFP (PX458) plasmid (Addgene) to obtain the two miR483-gRNA-plasmid constructs.

*Igf2*^+/+^ and *Igf2*^dGGCT^ C2C12 cells^19^ were seeded and incubated for 24 h prior to transfections. Transfections were done when the cells reached 80–90% confluency using JetPRIME transfection reagent (Polyplus transfection, BIOPARC, Illkirch-Graffenstaden, France). The constructs were transfected along with a linear puromycin marker (Clontech, Mountain View, California, United States) and the selection of stable cells was done by adding 1 µg/mL puromycin (Sigma) for 2 weeks. Cell culture media were changed every day during the first 3 days, and then whenever needed during the 2 weeks period. Control cells were obtained by following the same procedure but transfecting C2C12 cells with pSpCas9(BB)-2A-GFP (PX458) plasmid lacking any gRNA.

### miR483 mimics and inhibitors

The generated *miR483*^−/−^ cells and miR483^+/+^ control cells were seeded and transfected as described above. *miR483*^−/−^ cells were transfected with miR483-3p and miR483-5p mimics. Whereas miR483^+/+^ control cells were transfected with miR483-3p and miR483-5p inhibitors.

### DNA and RNA extraction

DNA was extracted using the DNeasy blood and tissue kit (Qiagen) and the concentration was measured using a NanoDrop ND-1000 spectrophotometer (Thermo Fisher Scientific). Total RNA was extracted using the RNeasy mini kit (Qiagen). Total RNA including miRNA was extracted using the miRNeasy mini kit (Qiagen). RNA concentration and quality were measured using Tape Station 2200 (Agilent Technologies). All RNA extractions were combined with DNase I (Qiagen) treatment.

### RT-qPCR

Total RNA and miRNA were converted to cDNA using the high capacity reverse transcription kit (Applied Biosystems, Foster City, California, United States), and the TaqMan advanced cDNA synthesis kit (Applied Biosystems), respectively. cDNA was used to perform quantitative PCR (qPCR) on a QuantStudio 6 Flex instrument using the QuantStudio Real-Time PCR software v1.3 (Applied Biosystems). The volume of the qPCR reactions was set to 10 µL.

### Proliferation assay

C2C12 cells with different genotypes were cultured in 6-well plates. Each cell clone was cultured in 16 wells (10^4^ cells/well) at day 0. Then at each day, for a total of 8 days, the number of cells were counted from two wells for each of the clones. Cell counting was performed by washing the cells with PBS, adding 0.5 mL trypsin for 3 min at RT before adding 1 mL of cell culture medium, which includes FBS, to deactivate trypsin. A volume of 12.5 µL of the cell mixture was mixed in a 1:1 ratio with Trypan blue, and counted on a Countess II Automated cell counter instrument (Invitrogen).

### RNA-Seq of cell lines

Total RNA was extracted from the developed stable cells as described above. The mRNA was selected using the Dynabeads mRNA DIRECT purification kit (Thermo Fisher Scientific). RNA-seq libraries were prepared using the SENSE Total RNA-Seq Library Prep kit (Lexogen, Campus-Vienna-Biocenter 5, Wien, Austria) and the libraries were amplified by a 17-cycle PCR. The quality and concentration of the prepared libraries were evaluated using TapeStation 2200 (Agilent Technologies). Equimolar amounts from each library were pooled and sent for sequencing at the SNP&SEQ platform (Uppsala University, Uppsala, Sweden) using the NovaSeq 6000 system (Illumina). A total of eight libraries, comprising one library per clone for each of the *Igf2*^+/+^_*miR483*^+/+^, *Igf2*^dGGCT^_*miR483*^+/+^, *Igf2*^+/+^_*miR483*^−/−^ and *Igf2*^dGGCT^_*miR483*^−/−^ cell lines, were sequenced which generated 35 to 50 million 50-bp paired-end reads per library.

### Bioinformatic analysis

Sequence reads were mapped to the reference mouse genome (mm10) using STAR 2.5.1b with default parameters^21^. HTSeq-0.6.1 (Python Package)^22^ was used to generate read counts. edgeR (Bioconductor package)^23^ was used to identify differentially expressed (DE) genes using gene models and an annotation GTF file from the mouse genome assembly mm10 (downloaded from UCSC genome browser, http://www.genome.ucsc.edu). The abundance of gene expression was calculated as count-per-million (CPM) reads. Genes with less than one CPM in at least two samples were filtered out. The filtered libraries were normalized using the trimmed mean of M-values (TMM) normalization method^24^. *P*-values were corrected for multiple testing using the False Discovery Rate (FDR) approach. For miRNA samples, one of the WT muscle samples was shown to be an outlier during the analysis and was therefore excluded. The miRNA annotation information was obtained from miRbase (downloaded 2018-03-05).

A gene ontology analysis of the DE genes were conducted using the Clusterprofiler R package^25^. All expressed genes were used as background, and the Biological Process and KEGG pathway tables were used to identify enriched GO terms. The gene set enrichment analysis (GSEA) was performed using the fgsea R package^26^. The genes were ranked based on the fold-change and the datasets were downloaded from the GSEA website (https://www.gsea-msigdb.org/gsea/downloads.jsp). The RNA-seq reads have been submitted to the sequence read archive (http://www.ncbi.nlm.nih.gov/sra) with the Bioproject accession number PRJNA744495.

## Results

### miRNA-seq from *Zbed6*-KO, *Igf2*-KI and wild-type mice

**Figure 1:**
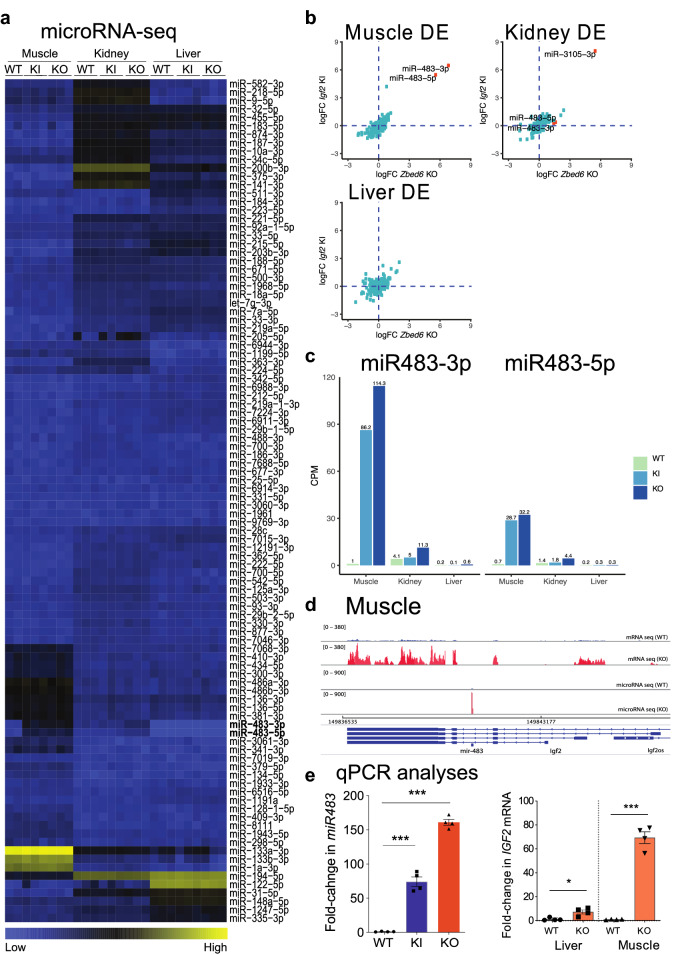
miRNA-seq analysis of mouse tissues in wild-type, *Zbed6*-KO and *Igf2*-KI mice. (**a**) Heatmap showing 100 expressed miRNAs across genotypes analyzed in each tissue separately, with miR483-3p and miR483-5p indicated in bold. (**b**) Scatterplots of log fold-change of miRNAs in *Zbed6*-KO and *Igf2*-KI mice compared with wild-type in muscle, kidney and liver tissues. (**c**) Expression of miR483-3p and miR483-5p in muscle, kidney and liver. The bars represent expression in counts per millions (CPM). (**d**) Uniquely mapped mRNA and miRNA reads at the *Igf2* locus in wild-type and *Zbed6*-KO muscle tissue. (**e**) qPCR analysis showing the expression levels of *miR483* in muscle tissue of WT, *Igf2*-KI and *Zbed6*-KO mice (left) and of *Igf2* mRNA in liver and muscle of *Zbed6*-KO mice (right). Graph shows the fold changes (mean ± SEM) compared to WT. *** corresponds to *P* < 0.001 in a two-tailed Student’s t-test.

To investigate whether the ZBED6 transcription factor controls miRNA expression, a miRNA-seq screen was performed using kidney, liver and skeletal muscle tissues from *Zbed6*-KO and WT C57BL/6 litter mates. Furthermore, because *Igf2* appears to be the most important downstream target for ZBED6^9^, we also performed a screen using our *Igf2*-KI mice in which the ZBED6 binding site in *Igf2* is disrupted. This experimental design allows us to dissect if an altered expression of a miRNA in *Zbed6*-KO mice is mediated through the interaction with its binding site in *Igf2* (Figs. 1 and S1).

The results show that ZBED6 has a minimal impact on regulating the expression of miRNAs in the tested tissues, with the exception of miR483-3p and miR483-5p that were upregulated in muscle and kidney tissue of *Zbed6*-KO mice (Figs. 1a–d and S1). A similar result was found in the *Igf2*-KI mice where miR483-3p and miR483-5p were significantly upregulated but only in muscle (Figs. 1a–d and S1) and another miRNA, miR3105-3p, showed upregulated expression in kidney (Figure S1). The up-regulation of miR483-3p and miR483-5p were validated by qPCR analysis (Fig. 1e, left). In liver, no significant differential expression was detected for any miRNA in any genotype comparison (Figure S1). In fact, neither miR483-3p nor miR483-5p were expressed at detectable levels (at least one read per million reads in at least two samples) in liver (Supplementary Table 2). qRT-PCR analysis confirmed the striking difference between muscle and liver tissue as regards the induction of *Igf2* mRNA expression when the ZBED6-*Igf2* axis is disrupted (Fig. 1e, right). RNAseq analysis assessing the expression of the three different *Igf2* transcripts further documents the dramatic upregulation of *Igf2* expression in skeletal muscle, heart and kidney in contrast to the minute upregulation in liver (Figure S2).

In conclusion, ZBED6 does not regulate miRNA expression in general, but ZBED6 inactivation or the disruption of its interaction with the *Igf2* locus results in a major upregulation of miR483 expression in some tissues including skeletal muscle but not in liver.

### Expression levels of *Igf2* and *miR483* in *Igf2*^dGGCT^ and *Igf2*^+/+^ C2C12 cells

**Figure 2:**
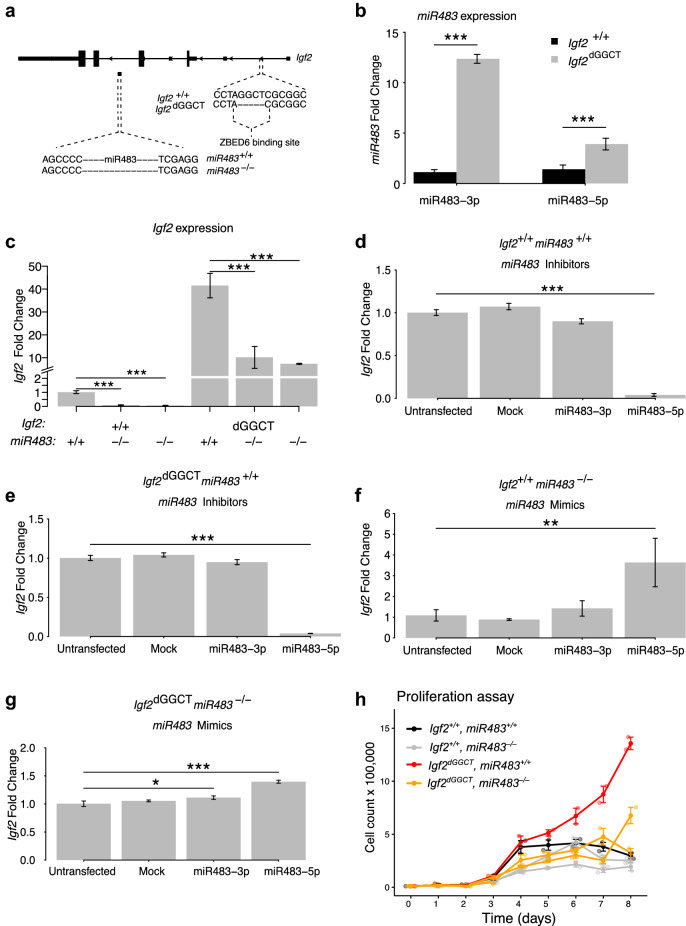
Disruption of *miR483* and its effect on *Igf2* expression in mouse C2C12 myoblast cells. (**a**) A depiction of the *Igf2* locus showing the *Igf2*^dGGCT^ mutation at the ZBED6 binding site, and the *miR483* sequences that were removed by CRISPR/Cas9. (**b**) qPCR analysis showing the expression levels of miR483 in *Igf2*^+/+^_*miR483*^+/+^ and *Igf2*^dGGCT^_*miR483*^+/+^ cells. (**c**) The expression levels of *Igf2* in *Igf2*^+/+^_*miR483*^+/+^, *Igf2*^+/+^_*miR483*^−/−^, *Igf2*^dGGCT^_*miR483*^+/+^ and *Igf2*^dGGCT^_*miR483*^−/−^ C2C12 cells. (D-G) The expression levels of *Igf2* in *Igf2*^+/+^_*miR483*^+/+^ (**d**), *Igf2*^dGGCT^_*miR483*^+/+^ (**e**), *Igf2*^+/+^_*miR483*^−/−^ (**f**) and *Igf2*^dGGCT^_*miR483*^−/−^ (**g**) cells transiently transfected with either inhibitors of miR483-3p and miR483-5p (**d**,**e**) or mimics of miR483-3p and miR483-5p (**f**,**g**). The ddCt method was used to normalize miR483 against miR15b-5p and *Igf2* against 18 s rRNA. *, ** and *** corresponds to *P* < 0.05, 0.01 and 0.001 in a two-tailed t-test, respectively. (**h**) Cell proliferation of wild-type and mutant C2C12 cells. Single clones were used for *Igf2*^+*/*+^*, miR483*^+*/*+^ and *Igf2*^*dGGCT*^, *miR483*^+*/*+^, while two clones each were used for *Igf2*^+*/*+^*, miR483*^*−/−*^ and *Igf2*^*dGGCT*^*, miR483*^*−/−*^. Each of the six clones was run in duplicates.

We previously established an *Igf2*^dGGCT^ C2C12 myoblast cell line using CRISPR^19^ which resulted in a deletion of four base pairs (GGCT) of the ZBED6 binding site in an intron of *Igf2* (Fig. 2a). Since *miR483* expression was upregulated in skeletal muscles of both *Zbed6*-KO and *Igf2*-KI mice (Fig. 1b,c), which both show more than 20-fold upregulated *Igf2* expression at the mRNA level in skeletal muscle^9^, a qPCR experiment was performed to test if the expression of *miR483* is also higher in *Igf2*^dGGCT^ compared to *Igf2*^+/+^ C2C12 cells. In fact, the qPCR results showed a 12-fold increase of miR483-3p expression and more than two-fold increase in miR483-5p expression in the *Igf2*^dGGCT^ cells (Fig. 2b). Furthermore, *Igf2* showed a 40-fold increase in mRNA expression in the *Igf2*^dGGCT^ cells compared to *Igf2*^+/+^ cells (Fig. 2c). Thus, the consequences of disruption of the ZBED6-*Igf2* interaction in C2C12 cells mimic the changes in *Igf2* and *miR483* expression in skeletal muscle in transgenic mice.

### Generation of *miR483*^−/−^ C2C12 cells

In order to further investigate the role of *miR483* in regulating *Igf2* expression in C2C12 cells, a guide-RNA was designed to target the *miR483* gene using the CRISPR-Cas9 method^27,28^. The plasmid was transfected into *Igf2*^+/+^ and *Igf2*^dGGCT^ C2C12 cells in an attempt to knock out *miR483* (Fig. 2a). Two *miR483*^−/−^ clones each were generated from *Igf2*^+/+^ and *Igf2*^dGGCT^ C2C12 cells.

RT-qPCR analysis showed that *Igf2* was significantly downregulated in the *miR483*^−/−^ clones compared to the *miR483*^+/+^ controls both on the *Igf2*^+/+^ and the *Igf2*^dGGCT^ background (Fig. 2c). The results confirm that *Igf2* expression is dependent on the expression of *miR483*^11^ most likely because miR483-5p stabilizes the *Igf2* transcripts^17^.

### miR483 inhibitors and mimics

*Igf2* expression was then analyzed in *Igf2*^+/+^ and *Igf2*^dGGCT^ C2C12 cells that were transiently transfected with miR483-3p and miR483-5p oligonucleotide inhibitors. Transient transfection of miR483-5p inhibitors caused a significant downregulation of *Igf2* expression compared to un-transfected cells and mock controls (Fig. 2d,e). Furthermore, the *miR483*^−/−^ clones were transfected with miR483-3p and miR483-5p mimics which resulted in a significant upregulation of *Igf2* expression in particular using miR483-5p (Fig. 2f,g), confirming the important role of miR483-5p for controlling *Igf2* expression in these cells.

### Disruption of *miR483* expression affects the proliferation of C2C12 cells

In order to evaluate the phenotypic effects of *miR483* downregulation, we measured the proliferation rate of the *miR483*^−/−^ C2C12 in *Igf2*^+/+^ and *Igf2*^dGGCT^ cells where the ZBED6-*Igf2* axis is disrupted. In comparison with the *Igf2*^dGGCT^_*miR483*^+/+^ cells, the knocking out of *miR483* from the *Igf2*^+/+^ and *Igf2*^dGGCT^ cells resulted a modest but statistically significant reduction in cell proliferation starting at days 3 and 5, respectively. Whereas, the *Igf2*^+/+^ cells in which miR483 was not knocked out showed a statistically significant reduction in cell proliferation only at day 8 (Fig. 2h). The result is in agreement with the altered expression of *Igf2* and *miR483* in the mutant cells as explained above (Fig. 2a–g), and shows that the marked upregulation of *Igf2* expression due to disruption of the ZBED6-*Igf2* axis in this cell line is dependent on the expression of *miR483*. (Fig. 2h).

### RNA-seq analysis

**Figure 3:**
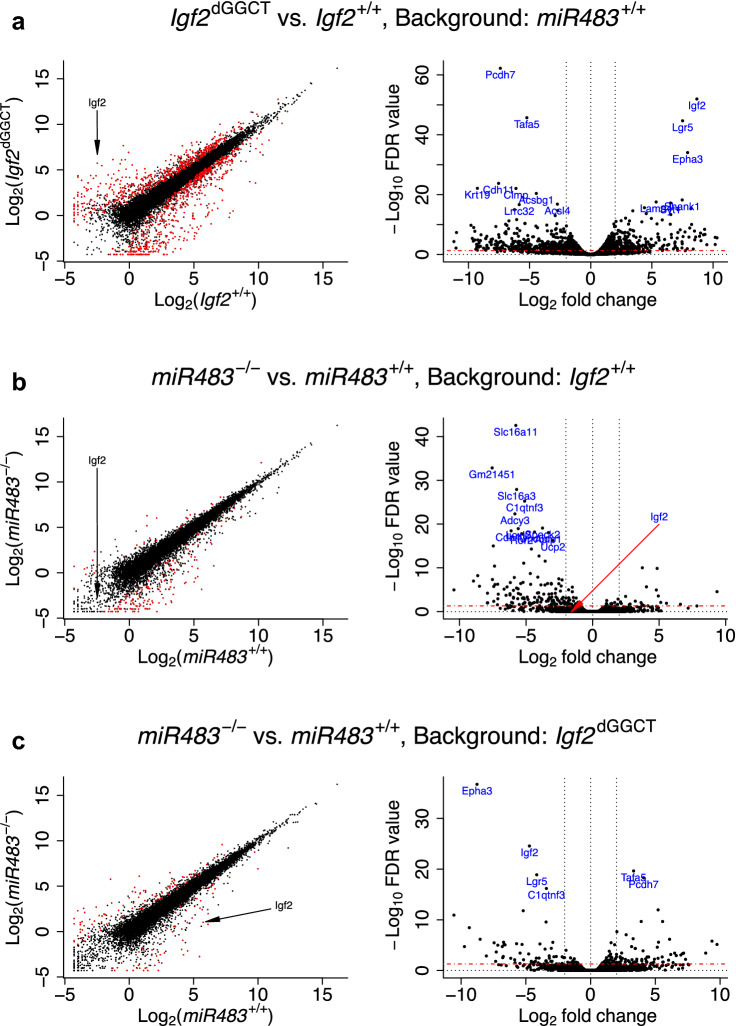
Results of RNA-seq analysis comparing *Igf2*^+/+^, *Igf2*^dGGCT^, *miR483*^+/+^ and *miR483*^−/−^ C2C12 cells. Scatter plots of RNA-seq expression levels in the log_2_ scale between the different contrasts (left), and volcano plots showing the log_2_ fold-change and the -log_10_ FDR values (right). Expression values are the averages of two samples per cell type. Statistically significant differentially expressed genes with log_2_ FC > 2 and -log_10_ FDR > 1.3 are plotted in red in the scatter plots, and -log_10_ FDR > 1.3 are indicated by a horizontal dashed red line in the volcano plots.

An RNA-seq analysis was performed using the *Igf2*^+/+^_*miR483*^−/−^ and *Igf2*^dGGCT^_*miR483*^−/−^ clones; the corresponding *miR483*^+/+^ clones were used as controls. The *Igf2*^dGGCT^/*Igf2*^+/+^ contrast performed on a *miR483*^+/+^ background showed almost a thousand statistically significant, differentially expressed genes (Figure S3, Supplementary File 2) with *Igf2* being one of the genes with the highest fold-change (Fig. 3a,b). Secondly, the *miR483*^−/−^/*miR483*^+/+^ contrast, performed on an *Igf2*^+/+^ background, showed only 182 differentially expressed genes with almost half of the genes being shared with the *Igf2*^dGGCT^/*Igf2*^+/+^ contrast (Figs. 3c,d and S3). Moreover, the *miR483*^−/−^/*miR483*^+/+^ contrast, performed on an *Igf2*^dGGCT^ background, showed only 164 differentially expressed genes with 76% of the genes shared with the *Igf2*^dGGCT^/*Igf2*^+/+^ contrast (Figs. 3e,f and S3). The expression of *Igf2* was found to be downregulated in the *miR483*^−/−^ cells compared with the *miR483*^+/+^ cells both on the *Igf2*^+/+^ and the *Igf2*^dGGCT^ background (Fig. 3c–f) in agreement with the qPCR analysis (Fig. 2c).

To investigate whether genes that are differentially expressed upon knocking-out *miR483* (background: *Igf2*^+/+^), are also differentially expressed in the *Igf2*^dGGCT^/*Igf2*^+/+^ contrast (background: *miR483*^+/+^), a scatter plot was made comparing the outcome of the two contrasts (Figure S4A). Out of the 182 differentially expressed genes that are significant in the *miR483*^−/−^/*miR483*^+/+^ contrast, 96 are significant in this contrast, but not in the *Igf2*^dGGCT^/*Igf2*^+/+^ contrast. However, almost half of these 96 genes (43 genes) showed a log_2_ fold-change greater than one in the *Igf2*^dGGCT^/*Igf2*^+/+^ contrast but did not reach statistical significance.

Similarly, the differential gene expression in the *Igf2*^dGGCT^/*Igf2*^+/+^ contrast (background: *miR483*^+/+^) and the *miR483*^−/−^/*miR483*^+/+^ contrast (background: *Igf2*^dGGCT^) were compared (Figure S4B). Out of the 164 genes that are significant in the *miR483*^−/−^/miR483^+/+^ contrast, 40 were not significant in the *Igf2*^dGGCT^/*Igf2*^+/+^ contrast although more than half of the genes (24 genes) showed a log_2_ fold-change value greater than 1 in the latter contrast.

These comparisons demonstrate that the 130 differentially expressed genes reaching statistical significance only in the two *miR483*^−/−^/*miR483*^+/+^ contrasts, contained 67 genes (49%) with at least a two-fold change in gene expression in the *Igf2*^dGGCT^/*Igf2*^+/+^ contrast, but the data did not reach statistical significance probably due to a limited statistical power. Moreover, the expression of 64 out of these 67 genes showed an opposite trend between *Igf2*^dGGCT^ cells, where *Igf2* expression is up-regulated, and *miR483*^−/−^ cells, where *Igf2* expression is down-regulated.

In conclusion, the considerable overlap in the genes showing differential expression in the *Igf2*^dGGCT^/*Igf2*^+/+^ contrast and in the two *miR483*^−/−^/*miR483*^+/+^ contrasts implies that an important biological function for *miR483* in these myoblast cells is its regulation of *Igf2* expression.

### Pathway analysis of *miR483*^−/−^ cells

**Figure 4:**
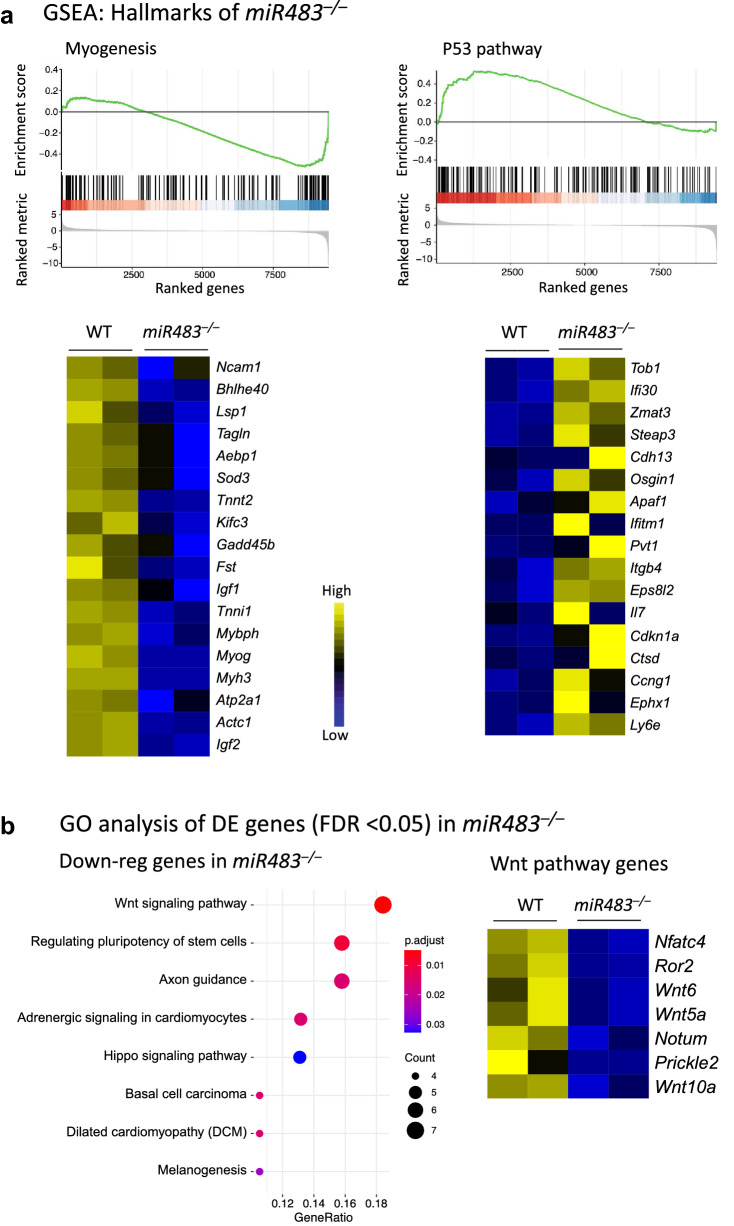
Pathway analysis of RNA-seq data comparing *miR483*^+/+^ and *miR483*^*−/−*^ C2C12 cells on an *Igf2*^+/+^ background. (**a**) Gene set enrichment analysis of ranked DE genes in miR483^−/−^ cells using hallmark gene sets (top) and heatmap of the genes contributing to the myogenesis or P53 pathway (below). (**b**) KEGG pathway analysis of down-regulated genes in *miR483*^*−/−*^ cells and heatmap of Wnt pathway-related genes.

In order to further explore the function of *miR483*, we performed gene set enrichment analysis (GSEA) and gene ontology (GO) analysis using the differentially expressed (DE) genes in *miR483*^−/−^ cells. The GSEA of ranked DE genes in *miR483*^−/−^ cells revealed a significant enrichment of myogenesis among the down-regulated genes, and a significant enrichment of genes involved in the P53 pathway among up-regulated genes (Fig. 4a, top). The heatmaps present the expression of the subset of genes that contributed the most to the enrichment signals, including *Igf2, Myog, Myh3* and *Tnnt2* as key down-regulated genes with a role in myogenesis and *Cdkn1a, Steap3* and *Tob1* as key upregulated genes in the P53 pathway (Fig. 4a, below). Furthermore, the GO analysis of significantly down-regulated genes (FDR < 0.05) in *miR483*^−/−^ cells showed a significant enrichment for the Wnt signaling pathway (Fig. 4b).

### The interaction between *miR483* and the ZBED6-*Igf2* axis

**Figure 5:**
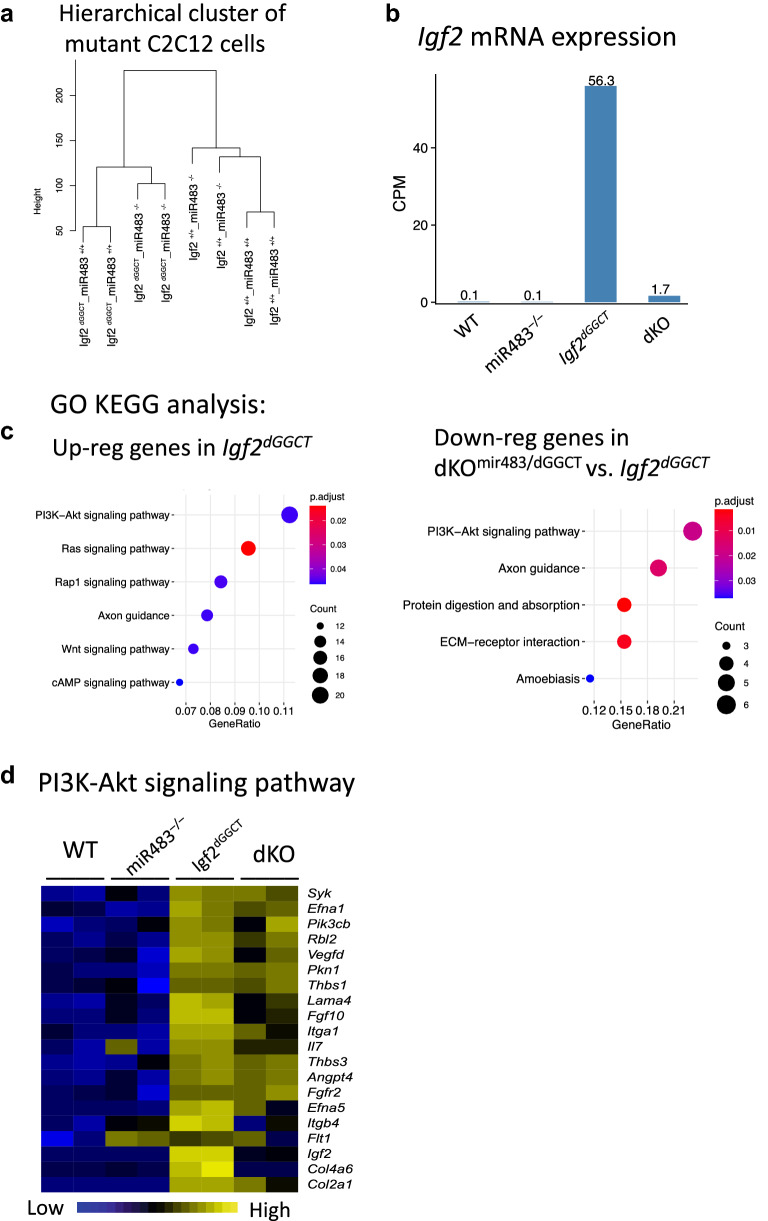
Comparison of RNA-seq data for wild-type, *miR483*^*−/−*^, *Igf2*^dGGCT^ and dKO C2C12 cells. (**a**) Hierarchical cluster of mutant C2C12 based on RNA-seq data. (**b**) Expression of *Igf2* as indicated by CPM values. (**c**) KEGG pathway analysis of DE genes in *Igf2*^dGGCT^ (left) and dKO^mir483/dGGCT^ cells (right). (**d**) Heatmap of genes found in the PI3K-Akt signaling pathway showing differential expression.

To gain further insight into the impact of *miR483* on the ZBED6-*Igf2* axis, we characterized the expression pattern in *Igf2*^dGGCT^ cells after knocking out *miR483*. Hierarchical cluster analysis of mutant C2C12 cells shows distinct differences between genotypes, with largest distance between *Igf2*^+/+^ and *Igf2*^dGGCT^ groups. Knocking out *miR483* from *Igf2*^dGGCT^ cells (dKO^mir483/dGGCT^) caused transcriptional changes resulting in an intermediate position of these cells in a cluster analysis including *Igf2*^+/+^ and *Igf2*^dGGCT^ cells (Fig. 5a). In agreement with this hierarchical clustering, the expression of *Igf2* was highest in *Igf2*^dGGCT^ cells, where the ZBED6-*Igf2* axis is disrupted, and moderate in dKO^mir483/dGGCT^ cells (Fig. 5b). The KEGG pathway analysis of DE genes in *Igf2*^dGGCT^ cells revealed a significant enrichment of the PI3K-Akt signaling pathway among the up-regulated genes (Fig. 5c,d). Remarkably, the opposite pattern was observed in dKO^mir483/dGGCT^ cells, where *miR483* is abolished, and the PI3K-Akt signaling pathway was enriched among the down-regulated genes (Fig. 5c,d). Taken together, these results further underscore the importance of an intact *miR483* function for the full effect of disrupting the ZBED6-*Igf2* interaction in C2C12 cells.

## Discussion

**Figure 6:**
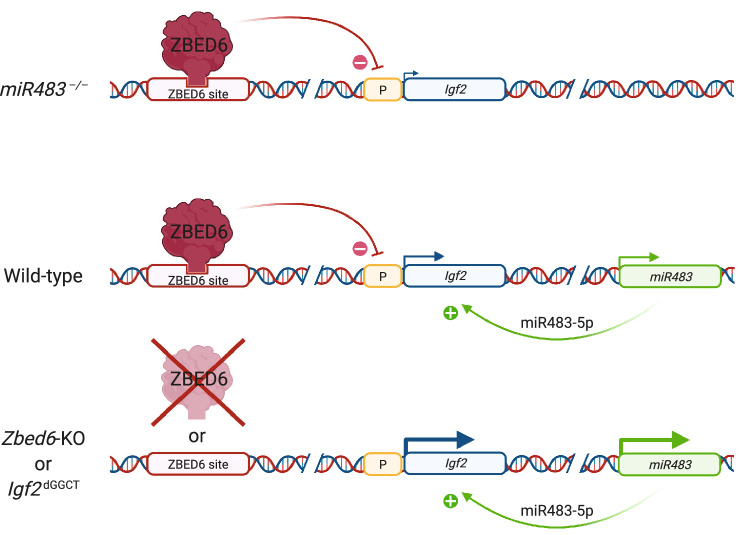
Schematic illustration showing the interaction between ZBED6 and the *Igf2*-*miR483* locus and how ZBED6 suppresses expression of *Igf2* and *miR483*, whereas miR483-5p enhances expression at the *Igf2*-*miR483* locus. The size of hooked arrows indicates level of expression. P, promoter. Figure created with BioRender.com.

The transcription factor ZBED6 is an innovation in placental mammals, and has evolved from a domesticated DNA transposon^4^. The extremely high sequence conservation (~ 100%) of the DNA-binding domain across species implies that it has an essential function in placental mammals^4^. A well-established function of ZBED6 is its regulation of *Igf2* expression after birth which has a strong impact on muscle growth and the size of internal organs^3,9^. The current study shows that it is not a regulator of miRNA expression in general but it suppresses the expression of *miR483* at least in muscle cells, as revealed by enhanced expression in *Zbed6*-KO and *Igf2*-KI mice, and in *Igf2*^dGGCT^ C2C12 cells (Fig. 6). The data for kidney cells is mixed since both miR483-5p and miR483-3p were upregulated in *Zbed6*-KO mice but not in *Igf2*-KI mice. The expression of miR483-5p and miR483-3p in liver was unaltered in both mouse models (Supplementary Table 2). These results combined with the well-established association between up-regulated expression of miR483 and tumorigenesis^11,12,16–18^ implies that disruption of the ZBED6-*Igf2* axis, either by impairing ZBED6 expression or blocking its access to the binding site in an intron of *Igf2*, can promote tumor growth in those cell types where ZBED6 suppresses *miR483* expression.

The analysis also revealed upregulated expression of miR3105-3p in kidney of *Igf2*-KI mice (Figure S1D), which is located at a distance of 0.5 Mb from a ZBED6 binding site identified by ChiP-seq analysis in mouse C2C12 cells^4^ and only 1.4 Mb from the *Igf2* gene on mouse chromosome 7. *Igf1r*, *Igf2r*, *Igf2bp1* and *Igfbp4* are all predicted to be potential targets of miR3105-3p^29^. But none of these predicted targets showed significant differential expression in RNA-seq analysis of mouse kidneys comparing *Zbed6*-KO and WT mice^9^. However, this does not necessarily mean that the proteins of these genes are not differentially expressed, as miRNAs can mediate translational repression^30^.

The present study shows that *miR483* is an important mediator for how ZBED6 regulates *Igf2* expression. ZBED6 suppresses *Igf2* expression partially by suppressing *miR483* (Fig. 6). This is illustrated by the finding that disruption of the ZBED6 binding site in *Igf2*^dGGCT^ C2C12 cells results in a ~ 40-fold upregulation of *Igf2* expression but this is reduced to an ~ eightfold upregulation when *miR483* is also deleted. Furthermore, our results show that miR483-5p, but not miR483-3p, enhances *Igf2* expression in mouse C2C12 cells, and this is consistent with the original report of this mechanism using human tumor cells^17^.

The data on C2C12 cells as well as our miRNA-seq data showed that ZBED6 has a major effect on regulating *miR483* expression in muscle cells, which in turn enhances the effect on *Igf2* expression. In contrast, *Zbed6* inactivation had no impact on *miR483* expression in liver (Figure S1E), a tissue where we did not detect *miR483* expression at all. Interestingly, a previous study in pigs showed that inactivation of the ZBED6 binding site in *Igf2* has a highly significant effect on *Igf2* expression in skeletal and cardiac muscle but not in liver^3,4^. It is possible that low expression of *miR483* in liver contributes to this lack of altered expression.

Our RNA-seq data comparing the transcriptome in *miR483*^+/+^ and *miR483*^−/−^ C2C12 cells indicate that a major function of *miR483* in this cell type is the regulation of *Igf2* expression because ~ 50% of transcripts showing differential expression between *miR483*^+/+^ and *miR483*^*−/−*^ cells also showed differential expression between *Igf2*^+/+^ and *Igf2*^dGGCT^ cells (Figure S3). This proportion was highest (76%) when *miR483* was knocked out on an *Igf2*^dGGCT^ background, where *Igf2* and *miR483* expression are enhanced. The pathway analysis of differentially expressed genes between *miR483*^−/−^ and miR483^+/+^ cells on an *Igf2*^+/+^ background showed a negative enrichment of myogenesis genes (Fig. 4a). This is in agreement with the known role of *Igf2* in myogenesis^19^. Interestingly, we found that modulating the expression of miR483 is sufficient to alter the downstream signaling pathway of IGF2. For instance, PI3K-Akt signaling pathway-related genes were found to be up-regulated in *Igf2*^dGGCT^ cells—where *Igf2* and *miR483* expression are enhanced—and down-regulated when *miR483* is deleted in the same cell type (Fig. 5c,d).

In summary, the present study introduces *miR483* as an important component of the ZBED6-*Igf2* axis that is required to regulate *Igf2* expression.

## Supplementary Information

Supplementary Information 1.

Supplementary Information 2.

Supplementary Information 3.

Supplementary Information 4.
