# Supplementary material for: ZBED6 regulates Igf2 expression partially through its regulation of miR483 expression
Source: Sci Rep. 2021 Sep 30;11:19484. doi: 10.1038/s41598-021-98777-0 (PMC8484269; doi:10.1038/s41598-021-98777-0)
Supplement: Supplementary file 1 — Supplementary Information 1. [file 41598_2021_98777_MOESM1_ESM.docx]

**Supplementary Tables**

**ZBED6 regulates *Igf2* expression partially through its regulation of *miR483* expression**

Rakan Naboulsi^1^, Mårten Larsson^1^, Leif Andersson^1,2,3,*^, Shady Younis^1,4,5,*^

^1^Science for Life Laboratory, Department of Medical Biochemistry and Microbiology, Uppsala University, SE-751 23 Uppsala, Sweden. ^2^Department of Animal Breeding and Genetics, Swedish University of Agricultural Sciences, SE-750 07 Uppsala, Sweden. ^3^Department of Veterinary Integrative Biosciences, Texas A&M University, College Station, TX 77843, USA. ^4^Division of Animal Breeding and Genetics, Ain Shams University, Shoubra El-Kheima, 11241 Cairo, Egypt. ^5^Division of Immunology and Rheumatology, Stanford University, Stanford, CA 94305, USA.

* To whom correspondence should be addressed.

Email: [syounis@stanford.edu](mailto:syounis@stanford.edu) or [leif.andersson@imbim.uu.se](mailto:leif.andersson@imbim.uu.se)

**Supplementary Table 1**. Names and sequences of the primers that were annealed and cloned into pSpCas9(BB)-2A-GFP (PX458) plasmid to establish the constructs that were used to knock-out *miR483*.

| Guide-RNA name | Sequence |
| --- | --- |
| mm-miR483_DSB-gRNA_1F | CACCGCAAGCCCAACCTCGGACCGT |
| mm-miR483_DSB-gRNA_1R | AAACACGGTCCGAGGTTGGGCTTGC |
| mm-miR483_DSB-gRNA_2F | CACCGAGCCCAACCTCGGACCGTGG |
| mm-miR483_DSB-gRNA_2R | AAACCCACGGTCCGAGGTTGGGCTC |

**Supplementary Table 2**. Read counts per million (CPM) of miR483-3p and miR483-5p, normalized with the weighted trimmed mean of M-values (TMM) method [1] in muscle, kidney and liver tissues of WT C57BL/6, *Igf2*-KI and *Zbed6*-KO mice.

| **Tissue** | **Type** | **miRNA** | **Normalized CPM** |
| --- | --- | --- | --- |
| Muscle | WT | miR483-3p | 1.75 |
| Muscle | *Igf2*-KI | miR483-3p | 81.62 |
| Muscle | *Zbed6*-KO | miR483-3p | 111.46 |
| Muscle | WT | miR483-5p | 1.75 |
| Muscle | *Igf2*-KI | miR483-5p | 27.48 |
| Muscle | *Zbed6*-KO | miR483-5p | 31.4 |
| Kidney | WT | miR483-3p | 4.07 |
| Kidney | *Igf2*-KI | miR483-3p | 4.95 |
| Kidney | *Zbed6*-KO | miR483-3p | 11.35 |
| Kidney | WT | miR483-5p | 1.39 |
| Kidney | *Igf2*-KI | miR483-5p | 1.83 |
| Kidney | *Zbed6*-KO | miR483-5p | 4.42 |
| Liver | WT | miR483-3p | 0.15 |
| Liver | *Igf2*-KI | miR483-3p | 0.09 |
| Liver | *Zbed6*-KO | miR483-3p | 0.58 |
| Liver | WT | miR483-5p | 0.16 |
| Liver | *Igf2*-KI | miR483-5p | 0.28 |
| Liver | *Zbed6*-KO | miR483-5p | 0.27 |

Reference

1. Robinson, M.D. and Oshlack, A., A scaling normalization method for differential expression analysis of RNA-seq data*.* *Genome biology.* **11**, R25 (2010).
