# Supplementary material for: ZBED6 regulates Igf2 expression partially through its regulation of miR483 expression
Source: Sci Rep. 2021 Sep 30;11:19484. doi: 10.1038/s41598-021-98777-0 (PMC8484269; doi:10.1038/s41598-021-98777-0)
Supplement: Supplementary file 2 — Supplementary Information 2. [file 41598_2021_98777_MOESM2_ESM.pdf]

Supplementary Information

ZBED6 regulates *Igf2* expression partially through its regulation of *miR483* expression

Rakan Naboulsi[1], Mårten Larsson[1], Leif Andersson[1,2,3,*], Shady Younis[1,4,5,*]

[1]Science for Life Laboratory, Department of Medical Biochemistry and Microbiology, Uppsala University, SE-751 23 Uppsala, Sweden. [2]Department of Animal Breeding and Genetics, Swedish University of Agricultural Sciences, SE-750 07 Uppsala, Sweden. [3]Department of Veterinary Integrative Biosciences, Texas A&M University, College Station, TX 77843, USA. [4]Division of Animal Breeding and Genetics, Ain Shams University, Shoubra El-Kheima, 11241 Cairo, Egypt. [5]Division of Immunology and Rheumatology, Stanford University, Stanford, CA 94305, USA.

* To whom correspondence should be addressed.

Email: syounis@stanford.edu or leif.andersson@imbim.uu.se

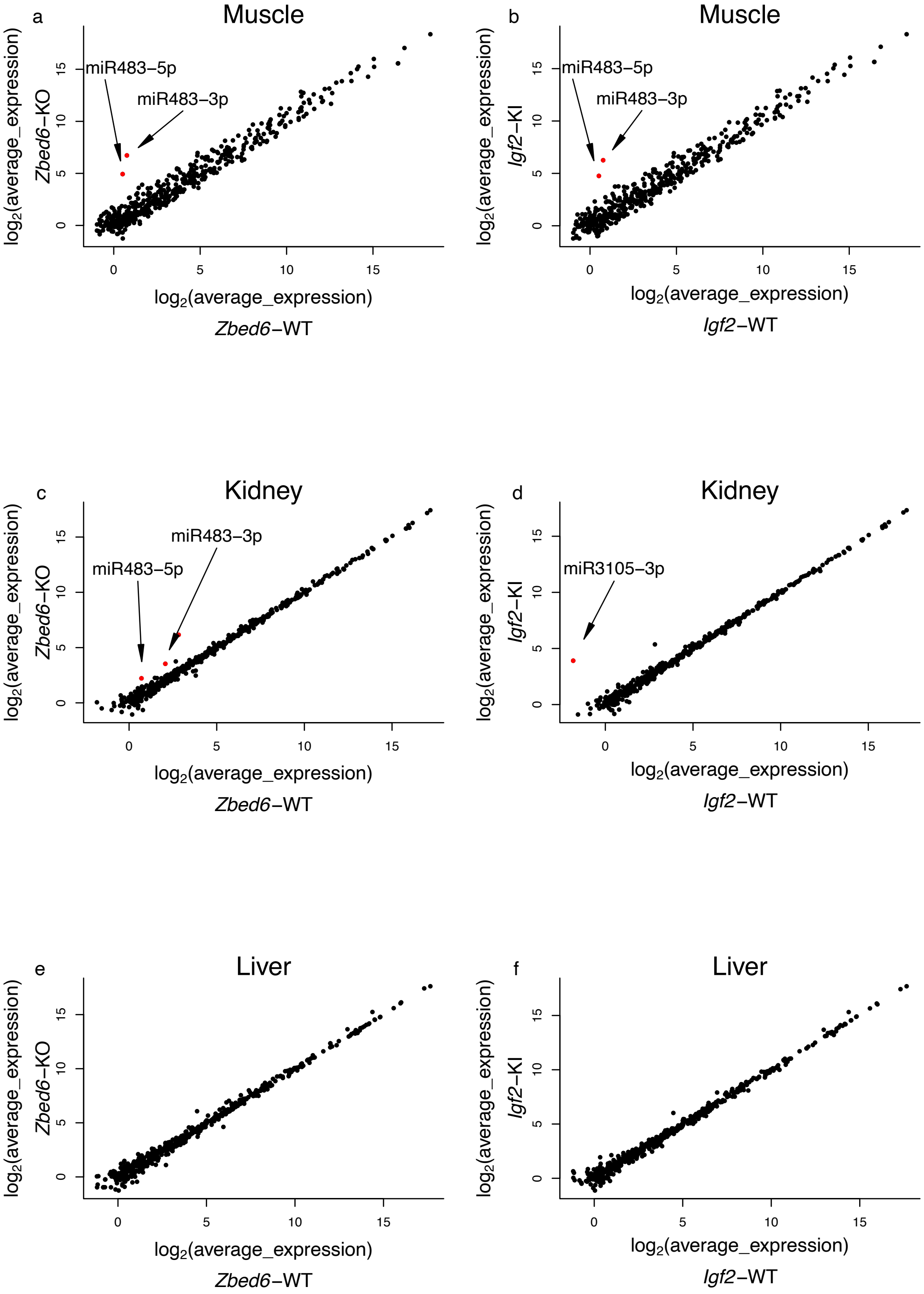

Supplementary Figure S1. miRNA-seq analysis showing the $\log_2$ expression of miRNA in skeletal muscle (A, B), kidney (C, D) and liver (E, F) from *Igf2*-KI, *Zbed6*-KO and wild-type C57BL/6 litter mate mice. Expression values are the average of three samples per tissue for each genotype. Statistically significant differentially expressed miRNAs were identified using the likelihood ratio statistical test.

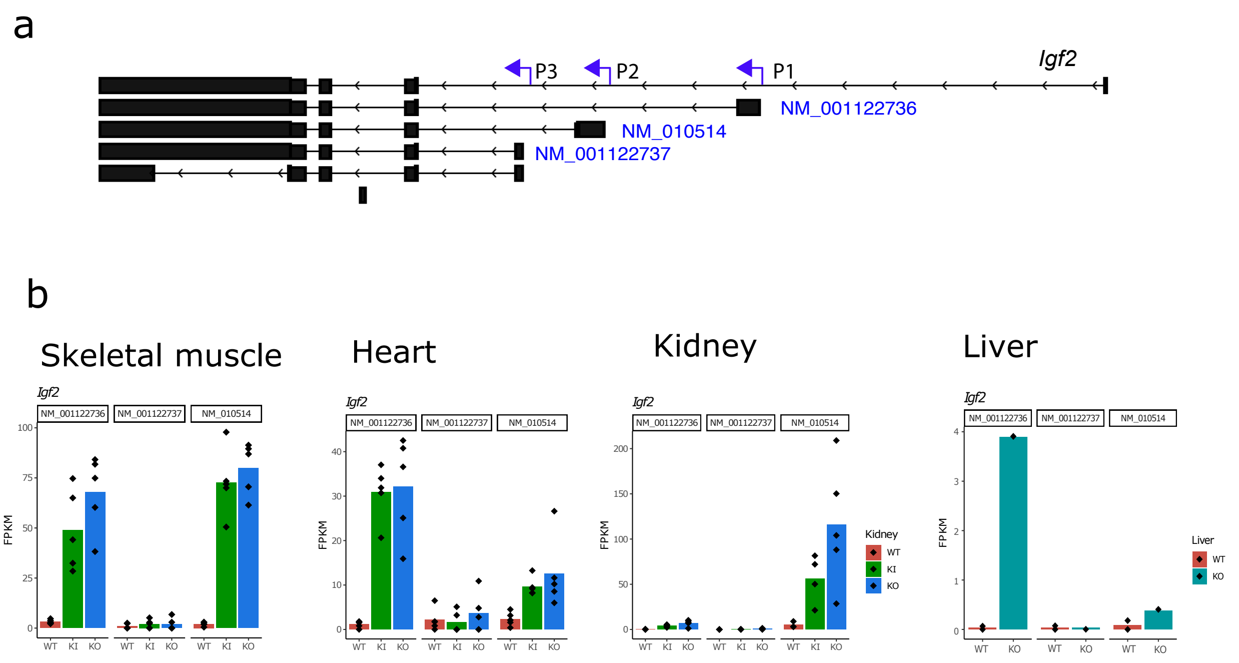

Supplementary Figure S2. *Igf2* expression in wild-type, *Zbed6*-KO and *Igf2*-KI mice (24-26 week of age) as determined using RNAseq analysis [1]. (A) Schematic illustration of the three transcripts from the P1, P2 and P3 promoters in mice, indicated by blue arrows. (B) Expression of three different *Igf2* transcripts in skeletal muscle, heart, kidney and liver. Note the low level of expression in liver in comparison with the other tissues.

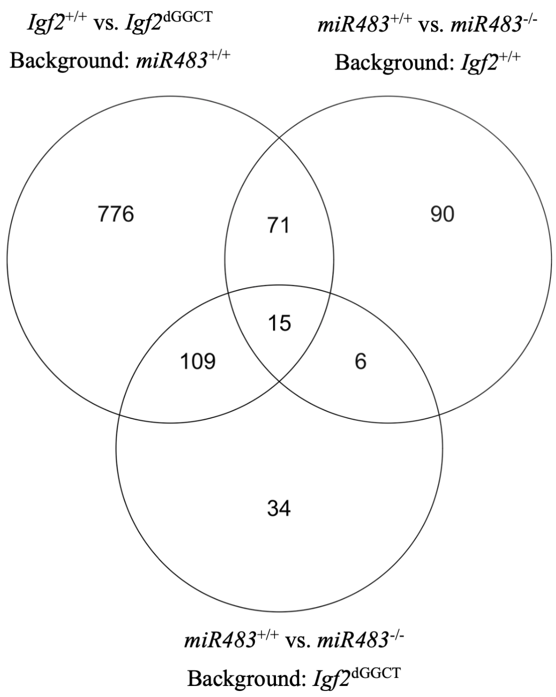

Supplementary Figure S3. Venn diagram showing the distribution of statistically significant differentially expressed genes with at least one-fold change in one or more of three contrasts of C2C12 cells with different genotypes.

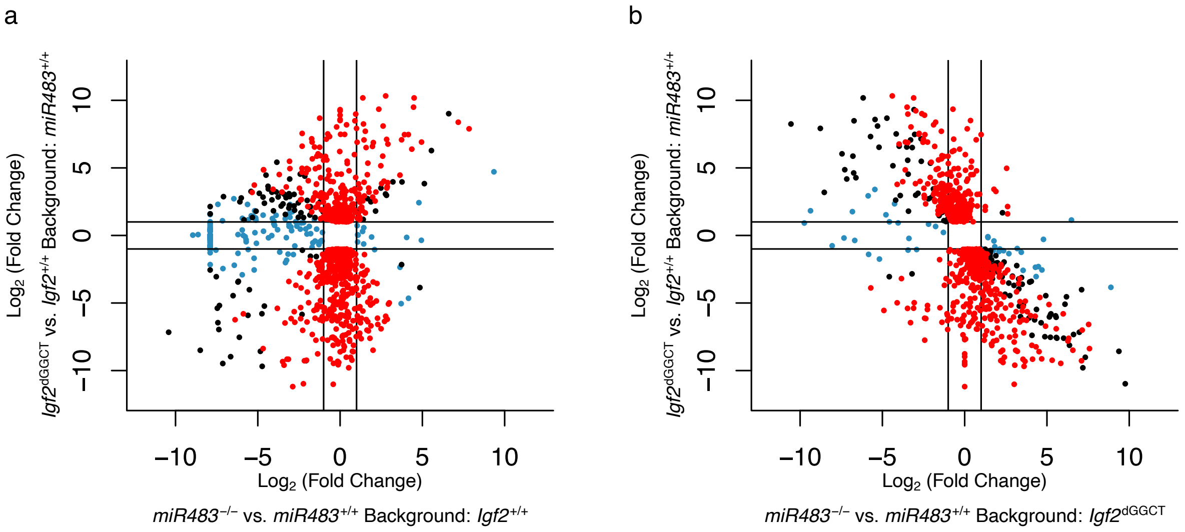

Figure S4. Scatter plots showing $\log_2$-fold changes in RNA-seq data. (A) Comparison of the *Igf2*^{dGGCT}/*Igf2*$^{+/+}$ contrast performed on a *miR483*$^{+/+}$ background and the *miR483*$^{-/-}$/*miR483*$^{+/+}$ contrast performed on an *Igf2*$^{+/+}$ background. (B) Comparison of the *Igf2*^{dGGCT}/*Igf2*$^{+/+}$ contrast performed on a *miR483*$^{+/+}$ background and the *miR483*$^{-/-}$/*miR483*$^{+/+}$ contrast performed on an *Igf2*^{dGGCT} background are plotted. Genes showing significant differential expression only in the *Igf2*^{dGGCT}/*Igf2*$^{+/+}$ contrast, only in the *miR483*$^{-/-}$/*miR483*$^{+/+}$ contrast or in both are plotted in red, blue and black, respectively.

Reference

1. Younis, S., et al., The ZBED6-IGF2 axis has a major effect on growth of skeletal muscle and internal organs in placental mammals. *Proc Natl Acad Sci U S A*. **115**, E2048-E2057 (2018).
